# Editorial: Violent relationships: acute and long-term implications

**DOI:** 10.3389/fpsyt.2023.1290094

**Published:** 2023-10-20

**Authors:** Julia Schellong, Judith Daniels, Susan Garthus-Niegel

**Affiliations:** ^1^Department of Psychotherapy and Psychosomatic Medicine, Faculty of Medicine, University Hospital Carl Gustav Carus, Technische Universität Dresden, Dresden, Germany; ^2^Department of Clinical Psychology and Experimental Psychopathology, Faculty of Behavioural and Social Sciences, University of Groningen, Groningen, Netherlands; ^3^Psychologische Hochschule Berlin, Berlin, Germany; ^4^Faculty of Medicine, Institute and Policlinic of Occupational and Social Medicine, University Hospital Carl Gustav Carus, Technische Universität Dresden, Dresden, Germany; ^5^Faculty of Medicine, Institute for Systems Medicine (ISM), MSH Medical School Hamburg, Hamburg, Germany; ^6^Department of Childhood and Families, Norwegian Institute of Public Health, Oslo, Norway

**Keywords:** intimate partner violence, COVID-19, witnessing trauma, child maltreatment, INVITE study, PTSD, relationship, mother-infant bonding

Relationships are crucial for human wellbeing and mental health. They play a vital role in human development, shaping individuals through interactions with their surroundings and culture. Healthy relationships have positive effects across generations, offering protection during stress and aiding in healing (1). Social support and integration also guard against harmful behavior and health issues.

Conversely, strained relationships heighten vulnerability, negatively impacting mental and physical health. Interpersonal violence is a major global cause of harm, straining healthcare and economies (2). Abuse, especially from caregivers, can also foster further violence and cruelty, with enduring consequences. Unhealthy relationship experiences alter attachment patterns and predict future victimization, necessitating further investigation into their interplay.

Relationships and their impact on life are multifaceted. To reflect this, this Research Topic aimed to gather a collection of articles that shed light on different aspects of the psychosocial and psychosomatic effects of dysfunctional relationships. What unites the articles is a focus on relationships and violence, although they differ in their selection of the human concerns affected by them. The aim is to broaden the horizons of researchers and clinicians and to encourage cross-fertilization between different disciplines.

The Research Topic illustrates how violence in relationships has detrimental effects on different aspects of life, and how these effects in turn impact the general wellbeing of the affected. Studying the precise mechanisms of these impacts can then lead to better understanding of the preferences, needs and barriers in prevention, care, and treatment (see Figure 1).

**Figure 1:**
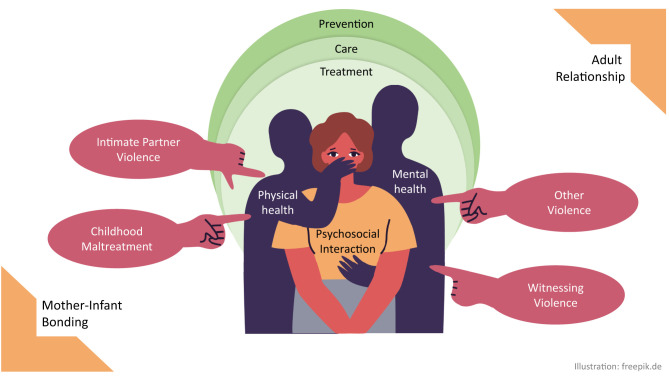
Impacts and needs in violent relationships.

This Research Topic includes seven articles. They range from systematic review (Thiel et al.) to primary research data (Wintermann et al.; Hitzler et al.; Frohberg et al.), study protocol (Seefeld et al.), therapeutic model (Mooren et al.), and the description of a model of the impact of witnessing violence (Trautmann et al.).

## Hidden violence in relationships: understanding IPV

In seemingly normal relationships, a disturbing reality often lurks beneath the surface—intimate partner violence (IPV). This hidden violence leaves lasting scars on victims, affecting physical and mental health. Amid COVID-19 lockdowns, rising anger and aggression were noted, with increased IPV risk. The systematic review by Thiel et al. supports the documented increase in IPV and underscores the need for accessible resources and awareness efforts in response. The INVITE study examines treatment preferences for intimate partner violence (IPV), depression, anxiety, and posttraumatic stress disorder (PTSD) among postpartum women in Dresden, Germany, at 3–4 months postpartum (Seefeld et al.). The study assesses differences in rates of IPV, postpartum depression, anxiety, and PTSD, along with treatment preferences between affected and non-affected women. Predictors of service preferences are also explored. The study's importance lies in informing tailored treatment and counseling services for postpartum women through understanding their needs and barriers.

## Enduring effects: the lasting impact of violent relationships

Violence within relationships does not necessarily fade with time. Childhood maltreatment (CM) can significantly impact mental health and wellbeing into adulthood, affecting various aspects of life. CM is linked not only to mental issues but also additional psychosocial risks that affect healthy development for both mothers and offspring. The study of Hitzler et al. reaffirms the association between CM and psychosocial risk factors in a dose-response manner. Vulnerability was particularly pronounced in postpartum women with a history of CM. To prevent negative consequences for mothers and children, early identification and support through evidence-based screening during pregnancy and postpartum are essential.

Postpartum psychopathology jeopardizes mother-infant bonding and child development. Frohberg et al. investigated how maternal adverse childhood experiences affects bonding via postpartum psychopathology, particularly in different forms of maltreatment. Findings highlight indirect effects on bonding, with emotional abuse having the strongest impact. Not the CM itself but the resulting psychopathology in postpartum mothers affects mother infant bonding. Remarkably, severe physical abuse correlated with better self-reported bonding. As a possible explanation, the authors name the desire of the women to compensate harmful relationships experienced as a child. This underscores the need to assess diverse maltreatment forms during the perinatal period for comprehensive insight.

Childhood maltreatment (CM) can potentially also impact the physical wellbeing. For example, psoriasis, a chronic inflammatory skin condition, might possibly be amplified by acute stress. Convergently, systemic therapies, including childhood trauma and stress assessment, show promise in managing psoriasis. To investigate the influence of childhood trauma and current stress on psoriasis, Wintermann et al. explored their roles in treatment outcomes among patients with moderate to severe psoriasis through a prospective cohort study.

## Witnessing trauma: unraveling the impact on relationships

When trauma unfolds before our eyes, relationships can become collateral damage. Traumatic events explicitly include not only events that are personally experienced but also events that are witnessed by an observer. Those who witness trauma, whether children or partners, can develop psychopathological reactions to events that are actually experienced by the beloved one. Acknowledging this impact is a step toward offering support and understanding to those affected. Risks exist at genetic, hormonal, and brain structure levels. Psychological and social factors also play a crucial role. Though frequently occurring, witnessing traumatic events, is understudied. What is unique about the study of Trautmann et al. is that it examines how specific trauma types are associated with distinct risk factors. The paper proposes that socio-cognitive and socio-affective mechanisms influence the processing and outcomes of witnessed trauma, thereby enhancing comprehension of such responses.

## Influence on wellbeing: relationships as agents of change

A growing body of research links parental trauma with psychosocial disorders, children's problems, and susceptibility to PTSD. Research reveals interconnected pathways involving relationships, psychology, and neurobiology, impacting generations. Parental trauma's impact extends within family dynamics and broader systemic contexts. The article of Mooren et al. examines intergenerational effects, guiding therapeutic interventions. A trauma-focused multi-family therapy is proposed, aiming to alleviate relational consequences, enhance resilience, and improve parent-child interaction. By nurturing emotional regulation and mentalization capacities, this approach benefits diverse families in culturally varied societies.

## Conclusion and future directions

Unfortunately, the editorial is limited by word constraints and therefore cannot delve into geographical or cultural variations that may influence the prevalence and impact of violent relationships. Additionally, while the Research Topic addresses violence primarily within heterosexual relationships, it is important to acknowledge that violent relationships can occur within diverse gender identities and orientations, which warrants further exploration. The summaries aim to present a condensed overview of the included articles and may not capture all nuances and variations in the research findings. Geographical and gender-specific perspectives could enrich the understanding of how violent relationships manifest and impact individuals in different contexts.

## Author contributions

JS: Conceptualization, Writing—original draft, Writing—review and editing. JD: Writing—review and editing. SG-N: Writing—review and editing.
